# Cone rod dystrophies

**DOI:** 10.1186/1750-1172-2-7

**Published:** 2007-02-01

**Authors:** Christian P Hamel

**Affiliations:** 1 Inserm U. 583, Physiopathologie et thérapie des déficits sensoriels et moteurs, Institut des Neurosciences de Montpellier, BP 74103, 80 av. Augustin Fliche, 34091 Montpellier Cedex 05, France

## Abstract

Cone rod dystrophies (CRDs) (prevalence 1/40,000) are inherited retinal dystrophies that belong to the group of pigmentary retinopathies. CRDs are characterized by retinal pigment deposits visible on fundus examination, predominantly localized to the macular region. In contrast to typical retinitis pigmentosa (RP), also called the rod cone dystrophies (RCDs) resulting from the primary loss in rod photoreceptors and later followed by the secondary loss in cone photoreceptors, CRDs reflect the opposite sequence of events. CRD is characterized by primary cone involvement, or, sometimes, by concomitant loss of both cones and rods that explains the predominant symptoms of CRDs: decreased visual acuity, color vision defects, photoaversion and decreased sensitivity in the central visual field, later followed by progressive loss in peripheral vision and night blindness. The clinical course of CRDs is generally more severe and rapid than that of RCDs, leading to earlier legal blindness and disability. At end stage, however, CRDs do not differ from RCDs. CRDs are most frequently non syndromic, but they may also be part of several syndromes, such as Bardet Biedl syndrome and Spinocerebellar Ataxia Type 7 (SCA7). Non syndromic CRDs are genetically heterogeneous (ten cloned genes and three loci have been identified so far). The four major causative genes involved in the pathogenesis of CRDs are *ABCA4 *(which causes Stargardt disease and also 30 to 60% of autosomal recessive CRDs), *CRX *and *GUCY2D *(which are responsible for many reported cases of autosomal dominant CRDs), and *RPGR *(which causes about 2/3 of X-linked RP and also an undetermined percentage of X-linked CRDs). It is likely that highly deleterious mutations in genes that otherwise cause RP or macular dystrophy may also lead to CRDs. The diagnosis of CRDs is based on clinical history, fundus examination and electroretinogram. Molecular diagnosis can be made for some genes, genetic counseling is always advised. Currently, there is no therapy that stops the evolution of the disease or restores the vision, and the visual prognosis is poor. Management aims at slowing down the degenerative process, treating the complications and helping patients to cope with the social and psychological impact of blindness.

## Disease name

Cone rod dystrophies (CRDs)

### Definition and diagnosis criteria

CRDs are inherited retinal dystrophies that belong to the pigmentary retinopathies group.

#### Functional signs and symptoms

• Decrease in the visual acuity is the earliest symptom

• Photophobia also occurs early

• Frequent dyschromatopsia

• Night blindness occurs later

#### Visual field

• Central scotoma appears first, preventing fluent reading

• Patchy losses of peripheral vision follow

• Severe loss of vision occurs earlier than in retinitis pigmentosa (RP)

#### Fundus

• Normal looking macula or fine macular lesions and pallor of the optic disc may be the only signs at early stage

• Pigmentary deposits resembling bone spicules, frequently in macular area

• Attenuation of the retinal vessels

• Waxy pallor of the optic disc

• Various degrees of retinal atrophy

#### Electroretinogram (ERG)

• Implicit time (between a- and b-wave peaks) shift at the 30-Hz flicker responses, along with delayed a- and b-wave single flash photopic response are early signs before amplitude reduction

• Dramatic decrease of amplitudes of both a- and b-waves

• Predominant involvement of photopic (cones) over scotopic (rods) responses

## Epidemiology

Prevalence of CRDs is estimated at 1/40,000 (thus, CRDs are ten times less frequent than RP) [1].

### Clinical description

#### Non syndromic cone rod dystrophies

CRDs present first as a macular disease or as a diffuse retinopathy with predominance of the macular involvement. In contrast to the symptoms of the rod cone dystrophies (RCDs, typical retinitis pigmentosa) resulting from predominant rod involvement, *i.e*. night blindness and loss of peripheral vision, the clinical signs of CRDs reflect the predominant involvement of cones, which leads to decreased visual acuity and loss of sensitivity in the central visual field. This fits the original description of the CRD entity in which cone loss precedes rod degeneration. However, in some cases, diffuse retinopathy affects simultaneously cones and rods, resulting in both night blindness and loss of visual acuity. These cases may also be considered as CRDs, although they overlap with other entities (see differential diagnosis). In general, CRDs are more severe than RCDs because the loss of patients' autonomy occurs earlier. It is convenient to describe two stages in the disease course of CRD.

**Figure 1:**
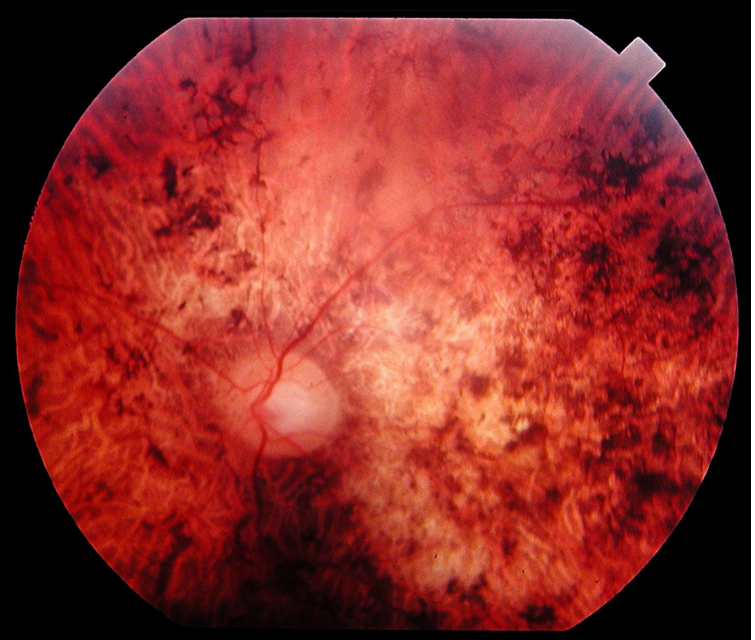
Fundus of a 45 year-old patient with cone rod dystrophy segregating with a loss-of-function mutation (E1087X) in *ABCA4*. Note the presence of various-shaped pigment deposits in the posterior pole with atrophy of the retina, while the retina appears less damaged in periphery (upper part of the photograph).

**In the first stage**, the main symptom is decreased visual acuity, which is usually discovered at school, in the first decade of life, and which does not significantly improve with spectacles. Patients often have a noticeable deviated gaze to project images on parafoveal regions of their retina that are less damaged. Along with this symptom, there are intense photophobia and a variable degree of dyschromatopsia. In contrast, night blindness is not mentioned by patients or, when present, is never as prominent as the decrease in visual acuity. Visual field testing shows central scotomas, while periphery is spared. As a result, patients have no difficulties to move. Fundus examination shows pigment deposits and various degrees of retinal atrophy in the macular region (Figure 1). Retinal vessels are usually normal or moderately attenuated. The optic disc is often pale at early stages, particularly on the temporal side, which accounts for the macular fibre bundle. At this stage, the question is to differentiate CRDs from macular dystrophies such as Stargardt disease, cone dystrophies and other rare macular conditions. Additional investigations help the diagnosis. First, fluorescein angiography and fundus autofluorescence show that the peripheral retina is also involved with heterogeneity in the fluorescence, but to a lesser extent than the macula. Second, the electroretinogram (ERG) shows a shift in implicit time of cone responses, followed by a decrease in both cone and rod responses. Cone responses are more severely affected than rod responses.

**In the second stage**, night blindness becomes more apparent and the loss in the peripheral visual field progresses. Therefore, patients have difficulties to move autonomously. In addition, visual acuity continues to decrease to a level where reading is no longer possible. Nystagmus is often present. At this stage, patients are legally blind (visual acuity <1/20), even though large parts of the peripheral visual field remain preserved.

#### Syndromic cone rod dystrophies

There are a few syndromes in which retinal degeneration characteristically features CRDs rather than typical RP.

**Figure 2:**
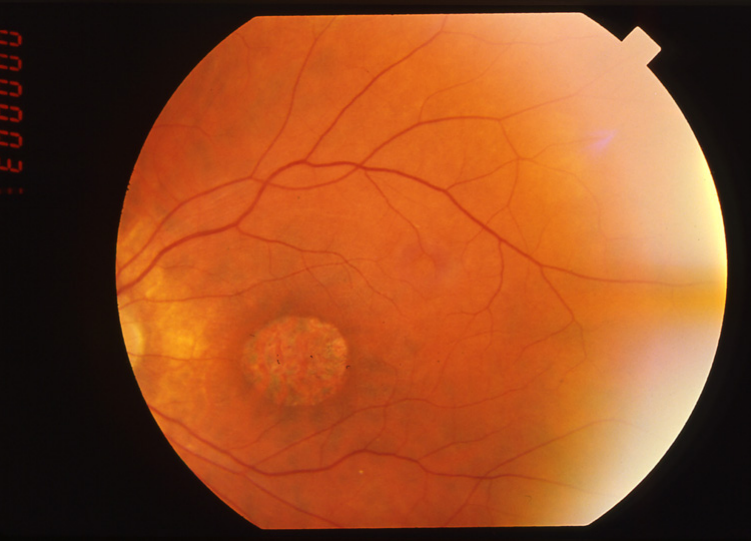
Fundus of a 31 year-old patient with Bardet Biedl syndrome. The peripheral retina does not show any large lesion but the macula is atrophic.

**Figure 3:**
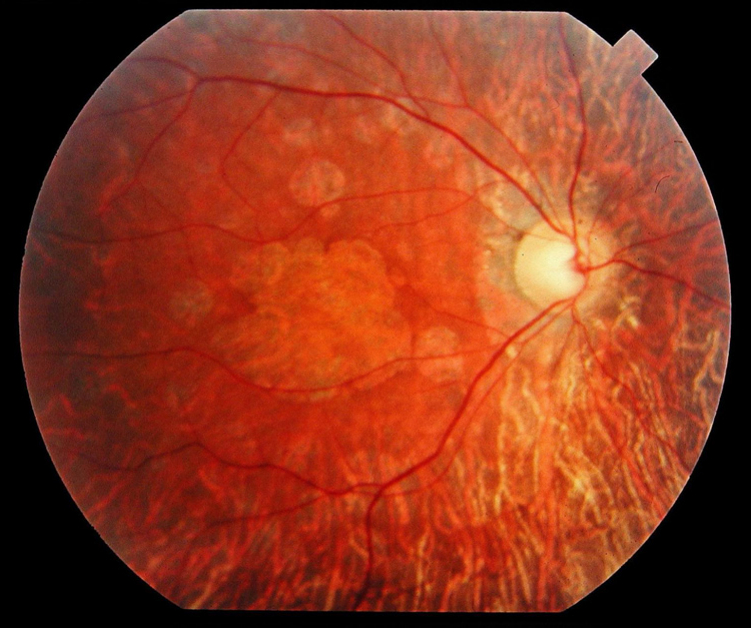
Fundus of a 34 year-old patient with cone rod dystrophy due to Spinocerebellar Ataxia Type 7 (SCA7). Note that the macular area, and also the mid periphery, are atrophic.

• **Bardet Biedl syndrome (BBS) **is an autosomal recessive disease with a prevalence ranging from 1/13,500 to 1/60,000. It associates retinal dystrophy with postaxial polydactyly, obesity, hypogenitalism, mental retardation or mild psychomotor delay, and renal abnormalities that can lead to renal failure. The retinal dystrophy is classically described as a RCD but many variants have been reported with a prominent macular involvement (Figure 2), indicating a CRD [2]. In fact, BBS patients have the diffuse type of CRD. In our experience, they always have macular involvement, with decreased visual acuity, photophobia and foveomacular hyperfluorescence on fluorescein angiography. The diagnosis of retinal dystrophy is often established in the first decade of life and legal blindness is reached before 20 years of age, but there are moderate forms of the disease. Diagnosis may be difficult when the clinical picture is incomplete. In this case, the presence of a CRD is an important sign. Twelve BBS genes encoding proteins involved in the cilium structure have been reported so far.

• **Spinocerebellar Ataxia Type 7 **is an autosomal dominant spinocerebellar degeneration due to expansions of polyglutamine in the ataxin protein. The retinal disease often begins with granular macula progressively spreading out to the whole retina, while the macula becomes atrophic (Figure 3) [3]. Initially, the disease often presents as an isolated retinal dystrophy; the characteristic macular involvement and the importance of visual impairment in a previously well seeing patient should lead to neurological investigations.

• **Ectodermal diseases**. CRD is sometimes encountered in:

○ *Amelogenesis imperfecta*. This refers to several conditions in which the tooth enamel is abnormal. One form of amelogenesis imperfecta with autosomal recessive inheritance (OMIM # 217080) is associated with CRD and abnormally shaped teeth [4-6].

○ *Hypotrichosis with juvenile macular *dystrophy [7]. It is a rare form of autosomal recessive alopecia associated with macular dystrophy. Usually, the retinal impairment is restricted to the macula but in a few instances it has been reported to be a CRD [8].

○ *Dysmorphic syndromes*. CRDs have been reported in spondylometaphyseal dysplasia [9] and in cleft lip [10].

○ *Metabolic dysfunctions*. CRD has been reported to occur in several metabolic disease (thiamine-responsive megaloblastic anemia [11,12]; one case with a mitochondrial mutation (T8993G) has been reported [13]. In addition, infantile Refsum disease with augmented phytanic acid and pigmentary retinopathy is associated with a characteristic prominent macular involvement, and in Alport syndrome (deafness, progressive nephritis) the fundus shows whitish flecks looking like crystals around the macula rather than an authentic pigmentary retinopathy.

### Etiology of non syndromic CRDs

Non syndromic CRDs are, like typical RP, genetically heterogeneous. Three Mendelian types of inheritance have been reported. Today, there are 13 genes responsible for non syndromic CRDs (10 cloned, 3 mapped). These genes can be classified in several categories.

***The 1st category ***includes genes mostly responsible for CRDs cases. The predominant one encodes the homeobox protein CRX, which controls rod and cone photoreceptor cell differentiation and survival. Most *CRX *mutations cause autosomal dominant CRD, with a prevalence estimated at 5 to 10% of dominant CRDs [14,15]. The severity of the disease is highly variable with some mild cases and some very severe cases. In fact, at this end of the spectrum, there have been a few reports of dominant Leber congenital amaurosis (LCA) caused by *CRX *mutations [16,17], a condition which is usually recessive, as well as a few RPs. Two other genes have been found only in CRDs. These are *RIM1*, reported in one family with autosomal dominant CRD [18] and *HRG4*, reported in one family with uncertain inheritance [19]. Interestingly, both encoded proteins are involved in photoreceptor synaptic transmission.

***The 2nd category ***includes genes mostly found in macular dystrophies. Today, it comprises essentially one gene, *ABCA4*, which is involved in the retinoid metabolism and causes the Stargardt disease. Mutations in the *ABCA4 *gene are responsible for 30 to 60% of the cases with autosomal recessive CRDs [20-25]. In some cases, the disease begins as a Stargardt macular dystrophy, which soon extends to the periphery. In other cases, the disease starts as a diffuse retinopathy with predominance of macular involvement. It has been shown that the *ABCA4 *mutations linked to CRDs are truncating mutations, often on both alleles, whereas amino acid change mutations are more frequently found in Stargardt disease. This suggests that more deleterious truncating mutations are associated with more severe conditions like CRD [26]. Belonging to this 2^nd ^category, mutations of *GUCA1A *have been described in one family with autosomal dominant CRD, while all other *GUCA1A *mutations are responsible for cone dystrophies. The *GUCA1A *gene encodes a protein that activates the guanylate cyclase (GC) [27]. GC itself is sometimes involved also in CRDs (see below).

***The 3rd category ***includes two genes mostly found in RP cases. One codes for the outer segment protein peripherin/RDS and is usually involved in autosomal dominant RP. It is well known that there are inter- and intra-familial phenotypic variations with *RDS *mutations including cases of dominant macular dystrophy or dominant CRD [28]. CRDs due to mutations in the *RDS *gene are relatively moderate in comparison with autosomal recessive CRDs, as the autonomy of patients is conserved in early adulthood. The other gene codes for RPGR (involved in opsin trafficking, particularly that of cone opsins). *RPGR *is the major causative gene for X-linked RPs, but it also accounts for some X-linked CRD with its locus referred as *COD1 *or *CORDX1 *[29] and cone dystrophies. As for RPs, CRDs caused by mutations in the *RPGR *gene are severe and diagnosed early in life. In addition to *RDS *and *RPGR*, the *CACNA1F *gene, whose mutations lead to X-linked congenital stationary night blindness, is mutated in one CRD Finnish family previously mapped as *CORDX3 *(or *COD4*) [30].

***The 4th category ***includes genes found in LCA. Today, there are three reported CRD families with mutations in the *RPGRIP1 *gene [31] (autosomal recessive inheritance) and *AIPL1 *[32] (autosomal dominant inheritance). These genes are usually involved in the pathogenesis of LCA. There are also several CRD families reported with mutations in *GUCY2D *[33,34], which is the major causative gene for LCA. In contrast to LCA patients, CRD patients with *GUCY2D*-mutations have a dominant condition, the mutations being restricted to exon 13 encoding the dimerization domain of the guanylate cyclase.

There are loci for which the gene remains to be cloned. For autosomal dominant CRDs, the *CORD1 *gene (undetermined localization) has been found in a Danish family with CRD and mental retardation [35]. *CORD4 *has been mapped in a Canadian family with CRD associated with neurofibromatosis [36]. For autosomal recessive CRDs, there are *CORD8 *mapped in a Pakistani family [37] and *CORD9 *in a Brazilian family [38]. For X-linked CRDs, *CORDX2 *has been mapped to Xq27 [39].

Taken together, it seems that most genes responsible for CRDs are involved in other types of retinal dystrophies, including RPs, macular dystrophies and cone dystrophies, thereby placing CRDs in the center of the vast panel of retinal dystrophies. One can therefore speculate that any gene causing retinal dystrophy may potentially be involved in CRD pathogenesis, and the challenge is to understand the underlying mechanisms. It seems clear that deleterious mutations of retinal dystrophy genes can cause very severe diseases, and hence CRDs. However, it is not yet clear why in some families, some members have macular dystrophy or RP, whereas other members (with the same mutations) have CRD. Likewise, the question why some mutations in a gene lead to CRD whereas others cause RP, remains unresolved for several genes.

## Diagnostic methods

Clinical diagnosis is based on the early decrease of visual acuity and photophobia, lesions in fundus, hypovolted ERG traces with predominant cone involvement, and progressive worsening of these signs. Full field ERG is the key test, particularly when patients are asymptomatic and show normal fundus at early stages. It is important to ascertain the diagnosis by repeating the examination one or two years after it has been first established. Multifocal ERG could be useful to follow precisely the functionality of the central retina.

At present, a systematic molecular testing is not routinely performed, due to the tremendous genetic heterogeneity of the disease. However, rapid and large-scale mutation screening techniques are developing and several laboratories perform search for mutations in the most frequently involved genes, including *ABCA4*, *CRX*, *GUC1A*; strategies to test in a short time several dozen of genes for a single patient DNA are emerging [23,40].

In some instances, molecular diagnosis for certain genes is performed by the laboratories that have discovered them.

### Differential diagnosis of non syndromic CRDs with other pigmentary retinopathies

CRDs are usually clearly differentiated from primary peripheral retinopathies and macular dystrophies. However, CRD may sometimes share features with several clinical entities.

#### Retinitis pigmentosa

• *Typical RP (rod cone dystrophy, RCD)*. In typical RCD, the diagnosis is easy because the first symptom is night blindness. This symptom typically remains isolated for several years with normal visual acuity before vision loss in daylight becomes prominent. In the fundus, pigment deposits are located in the periphery.

• *RP with early macular involvement*. In some cases, RCD has a typical slow progression but macular involvement occurs quite early, with some loss of visual acuity. A disease history characterized by predominant night blindness and prominent rod involvement on ERG supports the diagnosis of RCD.

• *Early onset RP or late stage RP*. In cases associated with early onset and severe RCD, the decrease in visual acuity with macular involvement may also occur early. It is again important to determine which sign, either night blindness or loss in central vision, appeared first in the disease course, and to perform ERG. The diagnosis may be particularly difficult when patients are examined at late stage. At that time, the typical changes in ERG are undetectable.

#### Leber congenital amaurosis (LCA)

This disease is associated with a high degree of visual impairment, which is already present at birth, and appears either as a rod- or cone-predominant disease, or both. Nystagmus, poor light fixation and reactivity, visual acuity lower than 1/20 and flat ERG are cardinal signs of the disease. Differential diagnosis with early onset CRD may be difficult because both diseases share the same clinical signs. The presence of a lapse time of several years before dramatic worsening of the visual disability will allow to classify the disease as CRD rather than LCA.

#### Maculopathies

Large, extended maculopathies may be difficult to differentiate from end stage CRD or RP. In all cases, the full field ERG is a key investigation.

• *Stargardt disease *is a maculopathy in which peripheral retina usually remains free of lesions. The disease is easy to recognize with the presence of yellow flecks that may cover the entire fundus (fundus flavimaculatus), hyperfluorescent macular lesions (bull's eye) and dark choroid on the fluorescein angiography. However, there are extended lesions in some late stage Stargardt cases, and in addition, a number of CRD are caused by the "Stargardt gene", *ABCA4*. In these cases, the early stage of the CRD may be similar to Stargardt disease, but, in a decade, signs of peripheral involvement occur.

• *Cone dystrophies*. Rods remain normal in these diseases. Main clinical signs are loss of visual acuity, photophobia, dyschromatopsia, and exclusive cone involvement at ERG. However, in some cone dystrophies, there may be some rod involvement, particularly in late stage. In contrast to CRDs, rods remain at least partly spared at these late stages, whereas they are non recordable in late stage CRD. Another sign is the absence of macular lesions for many years, even though the visual acuity is decreased.

#### Stationary retinal diseases

This is essentially achromatopsia, which is diagnosed on the basis of mainly cone involvement (rod being not entirely normal), the lack of disease evolution, and the normal fundus.

### Genetic counseling

Once the diagnosis CRD is made, patients should be informed and familial surveys recommended. Genetic counseling is always advised since all genetic forms can be encountered in CRD. A precise phenotypic diagnosis is always mandatory and is particularly useful in the absence of familial history or in sporadic cases.

### Antenatal diagnosis

Prenatal diagnosis can be performed in families in which the responsible gene has been identified. However, prenatal diagnosis (amniocentesis or chorionic biopsy) raises an ethical issue: whether the investigative risks associated with these invasive prenatal procedures are justified in a non life-threatening disease is questionable.

### Management including treatment

Currently, there is no therapy that stops the evolution of pigmentary retinopathies or restores the vision. However, there are several therapeutic strategies aimed at slowing down the degenerating process (light protection, vitaminotherapy), treating the complications such as cataract, macular edema, inflammation, and helping patients to cope with the social and psychological impact of blindness. In fact, management of CRDs is not different from the management of typical RP. A particular emphasis should be put on filtrating spectacles to minimize photophobia and on low vision aid. Patients are often severely visually disabled or legally blind by the end of the second decade of life. Therefore, it is important that their education focuses on an adapted professional occupation (teaching, computer based activities, physiotherapist).

### Unresolved questions

Cloned genes account for only a small part of the autosomal dominant CRDs cases and probably for half of the autosomal recessive cases. Therefore, genes remain to be discovered. Understanding the role of the encoded proteins often requires many years. Today, for a number of proteins, substantial information about their function is available, while some of the proteins remain poorly known.

A challenging issue is the elucidation of the precise steps leading from a gene mutation to photoreceptor degeneration. Data from animal models and clinical studies suggest that photoreceptors die by apoptosis at a linear rate throughout life (named the "one-hit hypothesis"), implying that they have a given probability to undergo apoptosis that remains constant from early to late stages of the disease [41]. For certain genes or severe mutations, this probability will be high, while for others it will be lower. The results of experimental and clinical studies clearly indicate that the mechanisms of photoreceptor degeneration are multiple. In all genetic forms of CRDs studied until now, data are incomplete. In addition, it is likely that several apoptotic pathways are involved in the photoreceptor loss, sometimes concurrently, and this also needs to be carefully investigated. This knowledge is crucial to design therapies. The efficacy of various potential treatments has to be proven in animal models and in humans. For example, gene replacement therapy for RDS in mouse improves photoreceptor ultrastructure, but there is no significant effect on photoreceptor cell loss [42].
